# Cooperative-PHD Tracking Based on Distributed Sensors for Naval Surveillance Area

**DOI:** 10.3390/s22030729

**Published:** 2022-01-19

**Authors:** Kleberson Meireles de Lima, Ramon Romankevicius Costa

**Affiliations:** PEE/COPPE—Department of Electrical Engineering, Federal University of Rio de Janeiro, Cidade Universitária, Centro de Tecnologia, Bloco H, Rio de Janeiro 21941-972, RJ, Brazil; ramon@coep.ufrj.br

**Keywords:** surveillance systems, tracking, PHD filter

## Abstract

Brazil has an extensive coastline and Exclusive Economic Zone (EEZ) area, which are of high economic and strategic importance. A Maritime Surveillance System becomes necessary to provide information and data to proper authorities, and target tracking is crucial. This paper focuses on a multitarget tracking application to a large-scale maritime surveillance system. The system is composed of a sensor network distributed over an area of interest. Due to the limited computational capabilities of nodes, the sensors send their tracking data to a central station, which is responsible for gathering and processing information obtained by the distributed components. The local Multitarget Tracking (MTT) algorithm employs a random finite set approach, which adopts a Gaussian mixture Probability Hypothesis Density (PHD) filter. The proposed data fusion scheme, utilized in the central station, consists of an additional step of prune & merge to the original GM PHD filter algorithm, which is the main contribution of this work. Through simulations, this study illustrates the performance of the proposed algorithm with a network composed of two stationary sensors providing the data. This solution yields a better tracking performance when compared to individual trackers, which is attested by the Optimal Subpattern Assignment (OSPA) metric and its location and cardinality components. The presented results illustrate the overall performance improvement attained by the proposed solution. Moreover, they also stress the need to resort to a distributed sensor network to tackle real problems related to extended targets.

## 1. Introduction

In 2015, the Brazilian Navy presented the strategic program, so-called the Management System of Amazônia Azul (SisGAAz). Its main objective is to monitor the surveillance of Brazilian jurisdictional waters and the international areas of responsibility for Search and Rescue (SAR) operations [1].

The SCUA (freely translated to the Unified Situational Awareness System) implements the SisGAAz’s first step in the gradual development of this Brazilian program. This system is a C4ISR (Command, Control, Communications, Computers, Intelligence, Surveillance, and Reconnaissance) that integrates multisensor and multisource data to either evaluate situational awareness or support decision-making activities for the maritime area of interest. In the context of situational awareness, multitarget tracking is a crucial task.

As for the problem of tracking multiple targets with an unknown number of targets, random finite sets have attracted significant attention since they were introduced in the 1990s by Mahler [2]. Since this new approach was introduced, several filters for its implementation have been proposed. In the 2000s, real-data implementations were emerging in areas as diverse as underwater active acoustics and air-to-ground GMTI (Ground Moving Target Indicator) detection and tracking [3].

Although the Probability Hypothesis Density (PHD) filter is already widely known, real-time implementation in systems that demand high performance and availability remains an issue. Over the years, several works have proposed ways to overcome these concerns. According to Gao et al. [4], although there is no data association problem in this filter, it can hardly be real-time implemented on a serial processor.

Different approaches have tried to overcome processing limitations or speed up processing. Zheng et al. [5] have proposed an efficient event-driven data particle PHD filter for real-time multi-target tracking. In order to reduce processing, the proposed method distinguishes the survival measurements, spontaneous birth measurements, and clutters for weight computation, which are steps with high computational complexity. With the same objective, Li et al. [6] present an algorithmic framework for parallel SMC-PHD filtering based on multiple processing. This algorithm fully parallels all Sequential Monte Carlo (SMC) steps.

Few papers address the parallelization and optimization of the implementation of SMC PHD filters. Li et al. [7] has proposed a novel Cardinality Consensus (CC) scheme to PHD filter for multitarget tracking in decentralized sensor networks with severely constrained communication. This scheme has a parallel filtering-communication mode as it performs communication parallel to filtering operations, which leads to data exchanges that require only a tiny amount of time. Li et al. [8] present details of GM PHD on the CC scheme, which is the first distributed GM filter based on the parallel filtering-communication mode, according to the authors.

In distributed multitargeting tracking applications, the fusion process of data received from sensors is another concern. Li et al. [9] propose a suitable real-time data fusion algorithm called Generalized Covariance Intersection (GCI). They, see [10], present another solution for this issue. This work introduces Fixed-nodes PHD (FN-PHD) fusion, which can achieve similar performance as PHD, but at much lower communication and computational costs. Firstly, radars track targets by a PHD filter. Then the target state closest to the fusion node is chosen, and all states among radars based on GCI are predicted and fused.

The SCUA applies parallelism to solve complex problems concerning data fusion in real time. The Brazilian Navy system has already implemented the PPTS (Pair of Plots in Two Stages) algorithm. This approach is a graph-based method proposed in [11], which is PP (Pair of Plot [12]) based. In 2015, Mello et al. proposed the implementation based on the model Gamma [13]. The proposed solution was the first parallel implementation of the PPTS method, which employed three Gamma models, where two of them exploited the resources of a parallel hardware environment (using MPI protocol and GPU).

This manuscript deals with the application of the GM PHD algorithm in a distributed sensor network, which can be: Radars or cameras installed along the coast or area of interest; radars on patrol or larger ships; and sensors installed in smaller boats. In such a system, unlike those presented in other applications [14,15,16], each node has its processing and tracking solution. Furthermore, through a communication link, they send their tracking data to a central station responsible for gathering and processing the information obtained by the distributed components.

The main contribution of this work is to propose an additional step of ‘prune & merge’ to the original GM PHD filter algorithm. This approach includes an additional step expecting to overcome the limitations of sensors and the original PHD algorithm. The proposed scheme presented good results attested by the OSPA metric in the simulations carried out. Furthermore, the target occlusion is overcome, which is one of the main weaknesses of the GM PHD algorithm.

The paper is organized as follows: Section 2 presents related works; Section 3 defines the MTT problem, presents the mathematical framework for RFS-based MTT, and presents the PHD filter definition; Section 4 illustrates a distributed network tracking system application, describes methods, presents the simulated scenarios and modeling, and analyzes the results; and Section 5 exposes the conclusions.

## 2. Related Works

Many algorithms and methods for tracking targets are available in the literature. Recursive filtering techniques under a Bayesian estimation framework are commonly used to solve the tracking problem using a stochastic approach. These algorithms and methods include extensions of the well-known Kalman Filter (KF): The Extended Kalman filter (EKF), Interactive Multiple-Model (IMM) filtering, and Variable-Structure IMM (VS-IMM) filtering, besides others filtering techniques, that include grid-based approaches, as well as nonparametric particle techniques [17].

The Kalman filter is the optimal estimator for stochastic white noise linear systems. When dealing with a nonlinear estimation problem, this technique has substantial performance limitations and no convergence guarantee. Extended and unscented KF variants circumvent the nonlinearity issues applying approximations. These algorithms are most effective when facing unimodal problems. The performance of Kalman Filter-based methods significantly decreases when applied to a multimodal problem, even in the most uncomplicated cases. It is precisely the problem considered in a maritime surveillance region: Monlinear models and multimodal distributions.

The Multitarget Tracking (MTT) problem is multimodal. An established way to overcome parametric methods limitations is achieved with the Multi-Hypothesis Kalman Filter (MHKF) or Particle Filter (PF)-based tracking. The objective of MTT is to jointly estimate, at each observation time, the number of targets and their trajectories from sensor data. Even at a conceptual level, MTT is a nontrivial extension of single-target tracking. Indeed, MTT is far more complex in both theory and practice [18]. According to [19], if the assumptions of the Kalman filter or grid-based filters hold, then no other algorithm can out-perform them. However, the assumptions in typical real scenarios do not hold, and approximate techniques are essentially employed. As mentioned by [20], the problems found in these applications favor and justify the use of MTT algorithms. The study by Jinan and Raveendran [21] presents a particle realization applied to MTT using the coordinate turn model to characterize targets dynamics in a two-dimensional maneuver representation. The survey by Wang et al. [22] introduces some advances in algorithms for multitarget tracking.

While the Kalman Filter is concerned with tracking a unique object under a single target problem, there are complementary steps of data association and management that compose multitarget tracking. Conventional MTT techniques typically employ divide-and-conquer approaches that partition a multitarget problem into a family of parallel single-target problems [23]. Given that each KF instance corresponds to a unique target, the data association issue arises as a consequence. If a wrong association occurs (i.e., some observation is associated with a wrong track), the system cannot recover from this error. When dealing with crowded scenes, it is not straightforward to assign an observation to a particular track [20]. One strategy to deal with this problem in Multiple-Hypothesis Tracking (MHT) is to delay data association decisions by keeping multiple hypotheses active until data association ambiguities are solved [24]. Data association is a process in which each measurement is hypothesized to have been originated from a known target, a new target, or clutter. This process is the vital point of MTT, and it becomes more complex with multiple targets, where the tracks compete for measurement [25]. For an introduction to conventional algorithms of data association in MTT, see [26]. In [27], the data association problem is treated as an optimization problem, and two methods are presented, one using a neural network and the other, an evolutionary algorithm.

Although these MTT techniques are well established, they present the problem of data association as an extra and even more complex difficulty. The lack of an optimal global solution for estimating target states and the absence of a Bayesian filter without some intermediate heuristic method exemplify such complexity. Alternatively, seeking to cope with this brittleness, Mahler [2] proposes an MTT algorithm based on the theory of Random Finite Sets (RFS). The indicated approach unifies into a single probabilistic procedure that contains detection, correlation, tracking, and classification. Finite-set Statistics (FISST) is the mathematical framework that supports the RFS approach, see [23,28,29] for a more detailed review.

Notwithstanding all RFS theoretical advantages, the RFS-based recursive Bayes filter, even in single-target problems, is so computationally challenging that it requires an approximate solution to make it implementable. For this purpose, Mahler [30] presents the PHD filter, which gives the number of expected targets when integrated over a region in target state space. Considering its recursive nature, by propagating PHD’s first-order moment statistics, it becomes computationally attractive. In their study of the PHD filter, Vo and Ma [31] propose an analytical solution for the PHD recursion of targets with linear dynamics. Clark and Bell [32] analyze convergence for Sequential Monte Carlo (SMC) approximation using a particle filter, while Clark and Vo [33] consider the Gaussian Mixture (GM) realizations under linear or nonlinear stochastic processes.

There are significant applications in the literature for the mentioned methods that demonstrate their use to multitarget tracking under a high clutter environment as [34,35,36,37] for SMC, and [38,39,40,41,42] for GM implementations. Different works [43,44,45] present the Cardinalized version (CPHD), which is a generalization of the PHD recursion, propagating jointly the posterior intensity and the posterior cardinality distribution, whilst PHD recursion propagates only the intensity. Mahler [46,47] introduces more advances in PHD approximations.

Regarding naval applications, Pace [14] implements SMC and GM PHD filter for a 3D radar applied to realistic three-dimensional aerial and naval scenarios. This work illustrates and compares their performance in detecting, initiating, and terminating tracks with clutter presence. Also related to the same type of application, Do et al. [16] propose a method for online tracking multiple targets for a naval area using a Generalized Labeled Multi-Bernoulli (GLMB) filter.

There is some research on surveillance and distributed sensors based on PHD filtering. However, few papers deal with maritime surveillance and distributed processing. Laneuville and Houssineau [48] consider the problem of multitarget tracking with passive data, obtained by geographically-distributed cameras using a GM-based algorithm. Battistelli et al. [49] treat CPHD applied to distributed multitarget tracking over a sensor network of heterogeneous and geographically-dispersed nodes with sensing, communication, and processing capabilities. Pailhas et al. [15] attempt Multiple-Input Multiple-Output (MIMO) sonar systems for area surveillance, especially to a harbor environment, with a restricted area located close to a traffic area, protecting it from underwater intrusion. Gulmezoglu et al. [50] investigate the use of GM PHD filters for multiple people tracking using a network of radar sensors in an indoor environment. Dias and Bruno [51] introduce a new cooperative particle filter algorithm for tracking emitters using Received-Signal Strength (RSS) measurements considering the communication’s cost. They propose two different solutions for reducing communication overhead with a modest degradation in performance. Concerning maritime surveillance, these cited applications have in common the following aspects: A central sensor fusion, stationary sensors, and no local PHD computation.

## 3. Background

This section provides minimal background to readers unfamiliar with the problem of tracking multiple targets, Bayesian filters, or the RFS approach.

### 3.1. Problem Statement

To formulate the tracking problem under a RFS framework, it is necessary to define some fundamental issues. According to Bar-Shalom and Li [52], estimation is the process of selecting a point from a continuous space—the “best choice” in line with some criteria; tracking is the state estimation of a moving object based on remote measurements, using one or more sensors at fixed locations or on moving platforms; and filtering is the current state estimation of a dynamic system—the reason for the use of the word “filter” is that the process for obtaining the “best estimate” from noisy data amounts to “filtering out” the noise.

In general, the objective of MTT is to jointly estimate, at each observation time, the number of targets and their trajectories from sensor data. Even at a conceptual level, MTT is a non-trivial extension of single-target tracking [18]. This problem has a two-fold objective: (1) Estimate the random number of targets that are present in the area of interest and (2) estimate the random state vector of each target [49].

Different from traditional MTT vector-based techniques that divide the problem into decoupled single-target problems, RFS tracking uses a state vector collection, treating the elements as random variables as well as the number of elements in that collection itself. Mathematically, the objective is to obtain the posterior multitarget joint probability density functions:

(1) $$p(X_{k},Z_{k}),$$

where $X_{k}$ and $Z_{k}$ are, respectively, the state and observations random finite sets, which are defined in the next section, at the instant *k*.

### 3.2. RFS Fundamentals

This section presents concepts and mathematical tools necessary to contextualize RFS-based tracking and Bayesian filtering. For a more detailed review, see [23,29].

A random finite set is a convenient probabilistic model for the representation of multiple stochastic dynamic systems (objects) and sensor measurements. Suppose, at the discrete-time *k*, there are $n_{k}$ objects with states $x_{k,1},\;\ldots ,\;x_{k,n_{k}}$ taking values in the state space $X\subseteq R^{n_{x}}$. Both the number of dynamic objects, $n_{k}$ and their individual states in X are random and time-varying. The multi-object state at instant *k*, represented by a finite set.

(2) $$X_{k}=\left\{x_{k,1},\;\ldots ,\;x_{k,n_{k}}\right\}\in F\left(X\right),$$

can be modeled as a random finite set on X. Here F(X) is the set of finite subsets of X [53].

Analogously, suppose that $Z_{k}$ is a measurement set from an observation process, which contains $m_{k}$ elements reported at time *k*, then:

(3) $$Z_{k}=\left\{z_{k,1},\;\ldots ,\;z_{k,m_{k}}\right\}\in F\left(Z\right),$$

is the RFS model on the observation space $Z\subseteq R^{n_{z}}$.

The cardinality (number of elements) and the individual state for a random finite collection are random variables that take values as unordered finite sets. These RFS characteristics provide this technique with the capacity to perform data association automatically and multitarget state estimation jointly.

The cardinality is a random variable and is modeled by a discrete distribution [54], according to:

(4) $$\rho \left(n\right)=Pr\left\{|X|=n\right\},$$

where n∈N, and Pr denotes the probability.

A random finite set is a finite-set valued random variable. Thereof, it has the usual probabilistic descriptors of a random variable in the sense of Finite Set Statistics (FISST), such as the Probability Density Function (PDF) and its statistical moments. The PDF of an RFS variable X is denoted f(X) and uniquely defined by the cardinality ρ(n) and symmetric joint distributions $f_{n}\left(x_{1},\ldots ,x_{n}\right)$. Mathematically, the FISST PDF definition is [54]:

(5) $$f\left(X\right)=f\left(\left\{x_{1},\ldots ,x_{n}\right\}\right)=n!\cdot \rho \left(n\right)\cdot f_{n}\left(x_{1},\ldots ,x_{n}\right).$$

The probability distribution of the cardinality itself is obtained according to:

(6) $$\rho \left(n\right)=\frac{1}{n!}\int f\left(\left\{x_{1},\ldots ,x_{n}\right\}\right)dx_{1}\ldots \;dx_{n}.$$

A central concept in FISST is the *set integral*. Given f(X) is a random variables distribution over a random set, the *set integral* of f(X) is defined by:

(7) $$\int f(X)\delta X=\sum_{n=0}^{\infty }\frac{1}{n!}\int f\left(\left\{x_{1},\ldots ,x_{n}\right\}\right)dx_{1}\ldots \;dx_{n}.$$

This integral characterizes the sum over the set cardinality, integrating all possible sets according to the number of elements. Besides, the term 1/n! considers the permutations of a set of size *n*.

The *intensity function* (also known as the probability hypothesis density or PHD) of an RFS X is defined as its first statistical moment [54]:

(8) $$D\left(x\right)=E\left\{\delta_{X}\left(x\right)\right\}=\int \delta_{X}\left(x\right)f\left(X\right)\;\delta X,$$

where $\delta_{w}\left(x\right)$ is the standard Dirac delta function concentrated at w and $\delta_{X}\left(x\right)$ is the set Dirac delta function, which is defined according to:

(9) $$\delta_{X}\left(x\right)=\sum_{w\in X}\delta_{w}\left(x\right).$$

It is important to indicate that the PHD is not a probability density. It is uniquely characterized by the following property. Given a region *S* of single-target state space, the integral is defined by:

(10) $$\hat{m}=\int_{S}D(x)dx,$$

and is the expected number of targets in *S*, i.e., equal to unity. In particular, if *S* is the entire state space (the multitarget state space), then the integral is the total expected number of targets in the scene [23]. An intuitive interpretation of this function considers it as the density of the expected number of targets occurring at a given point. Furthermore, the mass is seen as the density’s integral over the volume of space. Consequently, it is the expected number of targets. Additionally, the peaks of the intensity function indicate locations with a relatively high concentration of the expected number of targets, in other words, locations with a high probability of feature existence [55].

The Poisson RFS is a vital probability distribution type for this study. It is the unique RFS completely specified by its intensity function. Its name comes from the Poisson point process. If the RFS is Poisson, i.e., the number of points is Poisson distributed and the points themselves are IID (Independently and Identically Distributed), then the probability density of X can be constructed exactly from the PHD [55]. Summarily, its cardinality distribution, multi-object PDF, and intensity function follow, respectively, Equations (11)–(13).

(11) $$\rho \left(n\right)=\frac{e^{-\lambda }\lambda^{n}}{n!},\;n=0,1,2,\ldots$$

where λ>0 is the expected cardinality of X.

(12) $$f\left(X\right)=e^{-\lambda }\prod_{x\in X}\lambda \;p\left(x\right).$$

(13) D(X)=λp(x).

In this context, p(x) refers to the Probability Density Function (PDF) of a random variable x, which can be distributed as Gaussian or Uniform, for example.

### 3.3. PHD Filter Definition

A simple approach to set-based estimation is to exploit the physical intuition of the first moment of an RFS, known as its PHD or intensity function [55]. This approach is consistent with the multitarget equivalent of expected value—the expectation of an RFS. As stated previously, the PHD function corresponds to a mass density, and the mass corresponds to the expected value of targets in some state-space region S⊆X. For this reason, it is possible to link the PHD function and Bayesian framework.

In order to obtain a definition for the PHD function, there are two intuitive ways. The first one would be to obtain the PHD as a mathematical expectation of a point process density, as previously presented in Equation (8). The second one would be to treat the PHD as the limit of an occupancy grid probability.

As stated by Erdinc et al. [56], the surveillance region can be partitioned into bins, and it is assumed that each bin has the same volume. Furthermore, these bins are sufficiently small so that each bin is potentially occupied by at most one target [56]. Based on this, if $m_{i}$ denote the centre and $B(m_{i})$ the region defined by the boundaries of the *i*th grid cell, then the expected number of features over the region $S_{J}={⋃}_{i\in J}B\left(x_{i}\right)$ is given by:

(14) $$E\left\{\left|X\cap S_{J}\right|\right\}=\sum_{i\in J}P^{occ}\left(B\left(x_{i}\right)\right)=\sum_{i\in J}D\left(x_{i}\right)\lambda \left(B\left(x_{i}\right)\right),$$

where $X\cap S_{J}$ is the intersection between the state space X and surveillance area $S_{J}$, $P^{occ}\left(B\left(x_{i}\right)\right)$ denote the occupancy probability, $\lambda (B\left(x_{i}\right))$ is the area of the *i*th grid cell, and:

(15) $$D\left(x_{i}\right)=\frac{P^{occ}\left(B\left(x_{i}\right)\right)}{\lambda (B\left(x_{i}\right))}$$

is the intensity function. As the grid cell area tends to zero (or for an infinitesimally small cell), $B\left(x_{i}\right)\to dx_{i}$, the sum then becomes an integral, and the expected number of features in *S* becomes:

(16) $$E\left\{\left|X\cap S_{J}\right|\right\}=\int_{S}D(x)dx.$$

D(x) defines the PHD and it can be interpreted as the occupancy probability density at the point x [55]. The main objective for MTT RFS-based is propagating the intensity function to estimate targets state recursively based on the Bayesian framework.

Bayes theorem allows computing the probability of an event based on prior knowledge of conditions related to the event and some new evidence. This concept is the basis of Bayesian filters, which estimate the current state of a dynamic system in a probabilistic way. This process consists of two steps: Prediction and update, according to:

(17) $$p\left(x_{k}|z_{1:k-1}\right)=\int p\left(x_{k}|x_{k-1}\right)p\left(x_{k-1}|z_{1:k-1}\right)dx_{k-1},$$

and

(18) $$p\left(x_{k}|z_{1:k}\right)=\frac{p\left(z_{k}|x_{k}\right)p\left(x_{k}|z_{1:k-1}\right)}{\int p\left(z_{k}|x_{k}\right)p\left(x_{k}|z_{1:k-1}\right)dx_{k}},$$

where **x** is the state vector, and z is the observation vector, both in the conventional sense. Equations (17) and (18) are the recursive algorithm presentation of the Bayes Theorem.

Because of the Markov assumption, the posterior probability of the state is only determined based on the prior distribution, i.e., it is conditionally independent of the other earlier states. This premise is the basis for recursive estimation.

The presented filter has only conceptual importance. Because of the integral in Equation (17), it is computationally tractable only for a few discrete cases. However, all Bayesian recursive filters, such as Kalman Filter, are derived from it.

The recursive RFS filter is defined by Equations (19) and (20), similarly to the Bayesian filter one. Given the multitarget state $X_{k}$, and measurement $Z_{k}$ in the random finite set framework, suppose that at time k−1 the posterior FISST PDF of the multi-object state, $f_{k-1}\left(X_{k-1}|Z_{1:k-1}\right)$ is known. Here $Z_{1:k-1}\equiv Z_{1},\ldots ,Z_{k-1}$ is the sequence of all previous measurements. Then, respectively, the predicted and updated posterior multi-object densities is expressed as follows [53]:

(19) $$f_{k|k-1}\left(X_{k}|Z_{1:k-1}\right)=\int \Pi_{k|k-1}\left(X_{k}|X^{\prime }\right)f_{k|k-1}\left(X^{\prime }|Z_{1:k-1}\right)\delta X^{\prime },$$

(20) $$f_{k}\left(X_{k}|Z_{1:k}\right)=\frac{\varphi_{k}\left(Z_{k}|X_{k}\right)f_{k|k-1}\left(X_{k}|Z_{1:k-1}\right)}{\int \varphi_{k}\left(Z_{k}|X\right)f_{k|k-1}\left(X|Z_{1:k-1}\right)\delta X},$$

where $X^{\prime }$ is the set of targets previously observed, $\varphi_{k}\left(Z_{k}|X_{k}\right)$ is the observation likelihood function, and $\Pi_{k|k-1}\left(X_{k}|X^{\prime }\right)$ is the FISST transitional density (representing the multitarget motion model in the RFS sense). The posterior density is the estimate of a set of points from the multi-object filter at each time step. It is a target state estimate collection (unlabeled and unordered). Similar to the integral present in the Bayes filter, computing the exact multi-object posterior density is numerically intractable because of the set integrals. All the practical algorithms are approximations, and the PHD filter is one of these algorithms.

Mahler [30] derives a recursive Bayes filter for a PHD filter that accounts for multiple sensors, nonconstant probability of detection, Poisson false alarms, as well as the appearance, spawning, and disappearance of targets. The PHD is a best-fit approximation of the multitarget posterior in an information-theoretic sense and propagates the first-order statistical moment of the multitarget posterior.

For a Poisson point process, as described previously, the prediction and update recursive filter equations are [54]:

(21) $$D_{k|k-1}\left(x\right)=b_{k}\left(x\right)+p_{S}\int \pi_{k|k-1}\left(x|x^{\prime }\right)D_{k-1}\left(x^{\prime }\right)dx^{\prime },$$

(22) $$D_{k}\left(x\right)=D_{k|k-1}\left(x\right)\times \left[1-p_{D}+\sum_{z\in Z_{k}}\frac{p_{D}g_{k}\left(z|x\right)}{c_{k}\left(z\right)+\int p_{D}g_{k}\left(z|x\right)D_{k|k-1}\left(x\right)dx}\right],$$

respectively, where x is the state of a single object (a random vector), z is a measurement of a single object (a random vector), $b_{k}\left(x\right)$ is the PHD of the births at instant *k*, $p_{S}$ is the probability that a target still exists at time *k*, and $\pi_{k|k-1}$ is the target transition function, $D_{k-1}$ is the prior intensity, $D_{k}$ is the posterior intensity, $p_{D}$ is the probability that a target is detected at time *k*, $c_{k}\left(z\right)$ is the clutter PHD at time *k*, $g_{k}\left(z|x\right)$ is the target observation model. For simplicity, the probabilities of detection $p_{S}$ and $p_{D}$ presented here are independent of the state.

For computational implementations, see [38,39] for approximations based on Gaussian Mixture and [37,57] for particle methods.

This work implements a Gaussian Mixture realization. The GM is a weighted sum of Gaussians, according to:

(23) $$\mathrm{GM}\left(x\right)=\sum_{i=1}^{J_{k}}w_{k}^{\left(i\right)}N\left(x;m_{k}^{\left(i\right)},P_{k}^{\left(i\right)}\right),$$

where $w_{k}^{\left(i\right)}$ is the weight, $m_{k}^{\left(i\right)}$ is the mean, and $P_{k}^{\left(i\right)}$ is the covariance matrix of the *i*th component from total $J_{k}$.

For practical implementations, this algorithm needs complementary steps when compared to the canonical form of the Bayesian sequence. The GM PHD filter suffers from computational problems associated with the increasing number of Gaussian components as time progresses. This issue implies that the number of components in the posterior intensities increases without bound [38].

The prune & merge methods are the two most implemented methods to solve these issues in practical implementations. The first one consists of reaping components of the Gaussian mixture with values below a limit of *T*. Combined with the pruning operation, the merge method is applied to prevent unbounded growth. This method consists of clustering GM components in a region defined by the Mahalanobis distance *L* and delimited by threshold *U* according to:

(24) $$L:=\left\{i\in I=w_{k}^{\left(i\right)}>T|{\left(m_{k}^{\left(i\right)}-m_{k}^{\left(j\right)}\right)}^{T}{\left(P_{k}^{\left(i\right)}\right)}^{-1}\left(m_{k}^{\left(i\right)}-m_{k}^{\left(j\right)}\right)\leq U\right\},$$

where *j* refers to the component with the highest weight in set *I*. This distance measures how some points are distant from the mean, reflecting the sample dispersion considering the covariance matrix. An intuitive interpretation is that the more the data is correlated, the shorter the distance between them will be, forming a cluster.

The last step is the state extraction, which eliminates GM components with weak weights after the prune & merge step. A better alternative is to select the means of the Gaussians that have weights greater than an *Extraction threshold* *E* [38]. It is important to note that this step does not interfere with the filter performance.

Algorithm 1 presents the relationship between the filter equations and the execution steps for the GM PHD approximation.
**Algorithm 1***GMPHD*(*D*(**x**_*k*−1_))

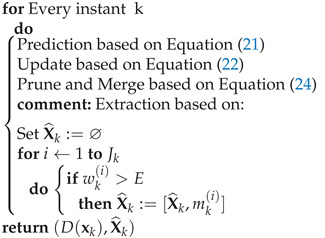



### 3.4. Optimal Subpattern Assignment (OSPA) Metric

The concept of error between a reference quantity and its estimated value plays a fundamental role in any filtering problem [58]. Unlike the idea of miss-distance in single-object tracking systems, such as the error between actual and estimated state, there is no direct way to measure this error in the multi-object case. As stated by Schuhmacher et al. [58], a satisfactory multi-object miss-distance needs to capture the “difference” between two sets of vectors, namely the reference multi-object state and the estimated multi-object state, in a mathematically consistent yet physically meaningful manner.

In short, the OSPA metric is the “distance” between a set of tracks and the known truths. This metric contains two error measures between those sets: Localization error component (accounts for state estimation) and cardinality error component (a benchmark for the number of missed targets). There is a third component out of the scope of this work called the labeling error component, which accounts for an incorrect assignment.

For two finite sets *X* and *Y* with respective *m* and *n* cardinalities, for m≤n, the OSPA distance metric is defined according to [58]:

(25) $${\bar{d}}_{p}^{\left(c\right)}\left(X,Y\right):={\left(\frac{1}{n}\left(\min_{\pi \in \Pi }\sum_{i=1}^{m}(d^{\left(c\right)}{\left(x_{i},y_{\pi (i)}\right)}^{p}+c^{p}\left(n-m\right)\right)\right)}^{\frac{1}{P}},$$

where $d^{\left(c\right)}\left(x,y\right):=min\left(c,d\left(x,y\right)\right)$ is the cutoff distance between two elements of *X* and *Y* with c>0 being the cutoff parameter, $\Pi_{n}$ represents the set of permutations of length *m* with elements taken from $\left\{1,2,\cdots ,n\right\}$, and 1≤p<∞.

The cutoff parameter *c* determines the relative weighting of the cardinality error component against the base distance error component. Larger values of *c* tend to emphasize cardinality errors and vice versa. The order parameter *p* controls the penalty assigned to “outlier” estimates (which are not close to the ground truth tracks). A higher value of *p* increases sensitivity to outliers [59].

## 4. Application for Tracking Maritime Surveillance

The SCUA integrates multisensor and multisource data to evaluate situational awareness and support decision-making activities for the maritime area of interest. Both types of sensors, shipboard or stationary, can belong to the distributed network. Therefore, different devices such as cameras, radars, and sonars can integrate the system.

For small ships, computers (sometimes IoT devices or tablets) have a limited processing capacity. System components exchange data via communication links such as 4G, LoRaWan, or satellite. As a result of processing capacity and bandwidth limitations, the tracker should have a low computational load. Allied to this, it should produce good results in a low clutter environment with a high probability of detection.

Each network member has a local application, which runs SCUA with several features and services, with the tracker being one of those. SCUA has parallel processes and computing (based on OpenCL), depending on the hardware used in the node. The parallelization method is not concerning in this manuscript.

**Figure 1:**
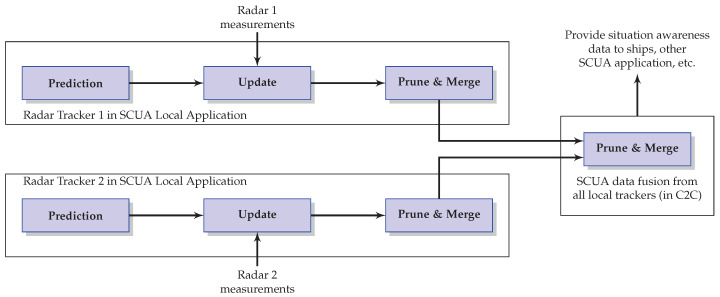
Probability Hypothesis Density (PHD) filtering and data fusion implementation scheme (example of two sensors available).

Figure 1 presents the implementation scheme for PHD filtering in local trackers and the data fusion in Central Station (C&C), for the case illustrated in this work. The simulations presented in this section use this same scheme.

### 4.1. Surveillance Scenario

Due to confidentiality issues, only simulations based on Matlab Sensor Fusion^®^ objects are used to illustrate the results of the application. In this simulation scenario, the distributed network is composed of two stationary sensors (2D radars). Both radars stare into the harbor, covering a 90-degree azimuth sector. The sensors are diagonally positioned in the surveillance area in opposite directions, according to Table 1. Each of them has a tracker responsible for monitoring targets in their respective FoV and providing the posterior intensity distribution.

This scenario was defined according to the main aspects: Availability and ease of access to data. There were no more sensors available at the collection and analysis of actual data to prepare the simulation. Furthermore, data obtained from sensors installed on ships could expose sensitive performance data. Given Brazilian regulations, this additional data would require significant effort to consult competent authorities. Despite this data limitation, this scenario proved appropriate for this work.

The effects of latency and bandwidth limitations in communication are not considered in the simulations. Due to the distances involved, the effect of the earth’s curvature in the observations is also not considered.

Additionally, there are vessels typically found in the maritime area of interest in simulation. There are five vessels in the harbor within the surveillance sector. Two of them are turning at 20 and 30 knots. The others are traveling with a constant heading of 10, 12, and 6 knots. Table 2 resumes information concerning the initial states of the ships.

**Figure 2:**
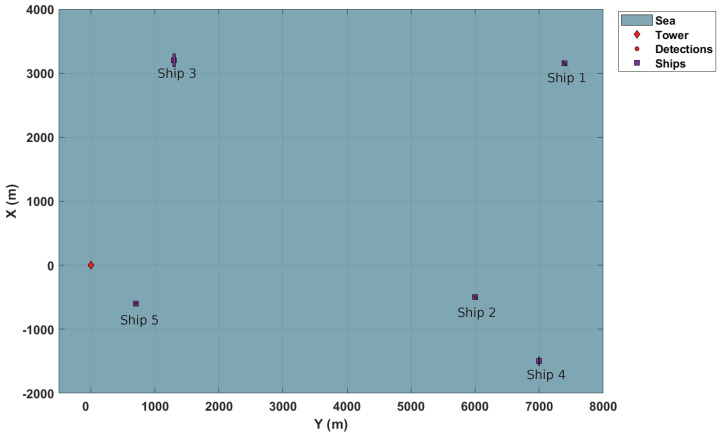
Maritime surveillance scenario.

Figure 2 illustrates the surveillance scenario described here.

### 4.2. Dynamic Modeling

Each of these five vessels in the simulation scenario has a kinematic state vector denoted by:

(26) $$x={\left[x\;\dot{x}\;y\;\dot{y}\;\omega \right]}^{T},$$

where (x,y) corresponds to position coordinates, $\left(\dot{x},\dot{y}\right)$ corresponds to velocities, ω is the constant turn-rate, and *T* denotes the transpose operation.

Another crucial dynamic model is the one used by the filter itself. Despite the problem of multitarget tracking, the dynamic model of a single target remains fundamental to establish the representative dynamics of each individualized target. Therefore, each target needs a motion model for the filter prediction step. The choice of this model is directly linked to the filter performance.

Tracking targets with coordinated turn motion is highly dependent on the models and algorithms [60]. Since the targets are possibly small, fast, and easy-to-maneuver vessels, the nonlinear nearly constant turn model is the best choice [61]. For a single target, the state dynamics is given by [38]:

(27) $$\begin{array}{cc} x_{k} & =F\left(\omega_{k-1}\right)x_{k-1}+Gw_{k-1} \end{array}$$

(28) $$\begin{array}{cc} \omega_{k} & =\omega_{k-1}+\Delta u_{k-1}, \end{array}$$

where $x_{k}={\left[x_{k}\;y_{k}\;{\dot{x}}_{k}\;{\dot{y}}_{k}\right]}^{T}$ corresponds to 2D position and velocities coordinates at instant *k*, ω stands for the turn rate in time *k*, $w={\left[w_{x}\;w_{y}\right]}^{T}$ is a zero-mean Gaussian process noise with $\sigma_{\omega }\in R^{2\times 2}$ covariance, *u* is a random variable with zero-mean, $\sigma_{u}$ is the covariance,

$$F\left(\omega \right)=\left[\begin{array}{cccc} 1 & 0 & \frac{\sin\omega \Delta }{\omega } & -\frac{1-\cos\omega \Delta }{\omega }\\ 0 & 1 & \frac{1-\cos\omega \Delta }{\omega } & \frac{\sin\omega \Delta }{\omega }\\ 0 & 0 & \cos\omega \Delta & -\sin\omega \Delta \\ 0 & 0 & \sin\omega \Delta & \cos\omega \Delta \end{array}\right],$$

$$G=\left[\begin{array}{cc} \frac{\Delta^{2}}{2} & 0\\ 0 & \frac{\Delta^{2}}{2}\\ \Delta & 0\\ 0 & \Delta \end{array}\right],$$

and Δ is the sample time.

For each target and stationary sensor, the observation model consists of azimuth and range measurements, according to:

(29) $$z_{k}=\left[\begin{array}{c} \sqrt{{\left(x_{k}-x_{s}\right)}^{2}+{\left(y_{k}-y_{s}\right)}^{2}}\\ \arctan\left(\frac{y_{k}-y_{s}}{x_{k}-x_{s}}\right) \end{array}\right]+\epsilon_{k}$$

where $\left(x_{k},y_{k}\right)$ is the target position at instant *k*, $\left(x_{s},y_{s}\right)$ is the sensor position, and $\epsilon_{k}$ is a zero-mean Gaussian process noise with $R_{k}\in R^{2\times 2}$ covariance.

### 4.3. Filter Parameters

The Gaussian Mixture PHD filters used in the simulations are that described in Equations (21) and (22). Each of them enables us to estimate the positions of the ships individually, as described before. Since the predictive model is nonlinear, the filter is the EKF version (see [38] for details about the Jacobians). Table 3 presents the filter parameters used in simulations.

For simulation purposes, there is no spawning, and the spontaneous birth RFS is Poisson, i.e., added uniformly inside the sensor’s field of view, with a rate of $10^{-5}$. The births have a unitary initial weight, and an initial covariance matrix:

$$P_{birth}=\left[\begin{array}{ccccc} 10^{5} & 0 & 0 & 0 & 0\\ 0 & 10^{5} & 0 & 0 & 0\\ 0 & 0 & 10^{5} & 0 & 0\\ 0 & 0 & 0 & 10^{5} & 0\\ 0 & 0 & 0 & 0 & 1000 \end{array}\right].$$

Finally, the clutter RFS follows the uniform Poisson model over the sensor FoV, the surveillance region being [−π/2,π/2] rad ×[0,12] km.

Since each radar independently estimates the coordinates of the targets, this information needs to be gathered. Then, the simulated central processing merges these distributions, obtaining the final solution in a distributed and cooperative way. Similarly to [62], the merging process is:

(30) $$\mathrm{Cooperative}\;\mathrm{Tracking}=merge(\left\{D{\left(x\right)}^{1},D{\left(x\right)}^{2},\ldots ,D{\left(x\right)}^{R}\right\}),$$

where $D{\left(x\right)}^{i}$ is the intensity estimated by *i*th distributed sensor, and *R* is the number of distributed sensors. Merge operation is a clustering technique. For RFS applications, the algorithms based on Mahalanobis distance are the most common method, which is detailed in [38].

**Figure 3:**
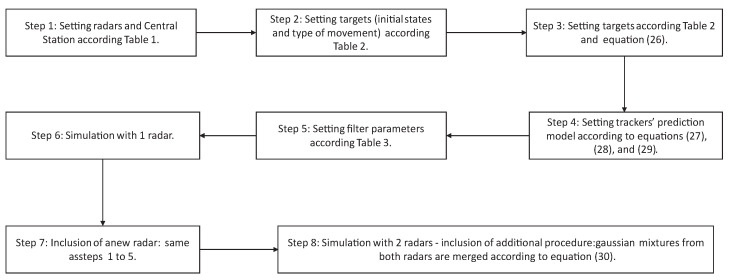
Block diagram of the proposed scheme.

Figure 3 resumes, in a block diagram, all the steps of the simulation setup (surveillance scenario, models, and filter parameters).

### 4.4. Results and Discussions

**Figure 4:**
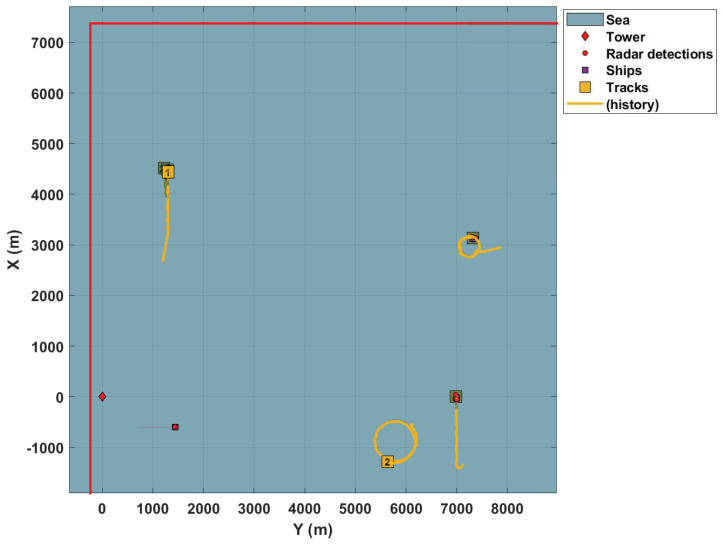
Tracking with one sensor.

**Figure 5:**
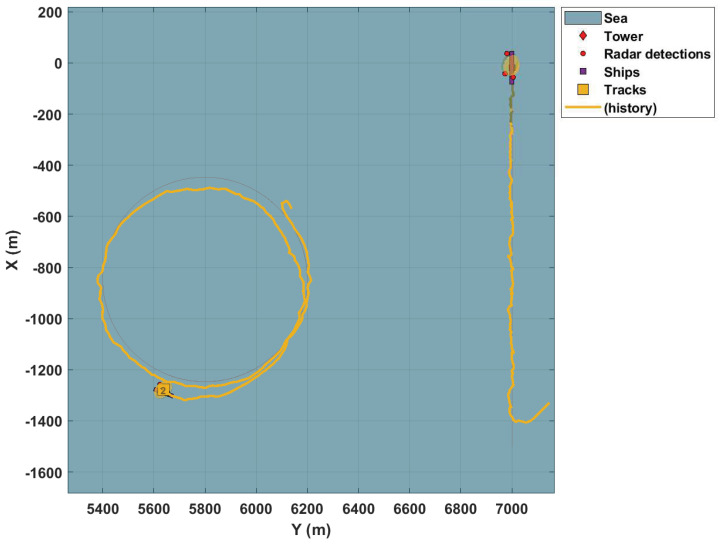
Tracking of two targets with different trajectories.

Based on OSPA metrics shown in Figure 10, the results presented in the simulations are satisfactory. Initially, to illustrate the performance achieved, the simulated tracking system uses only one radar, whose FoV is represented by the region delimited by red lines (as in the other figures). The tracker exhibits good results, i.e., it can track the targets observed by the sensor. Figure 4 and Figure 5 present these results.

As stated before, these figures demonstrate the tracking system’s performance with just one sensor. In addition, these figures show that the ships are tracked regardless of whether the movement performed is linear or circular, despite an initial transitory. It is noteworthy that the vessels present significant differences in their speed and movements. At the same time, one navigates at 12 knots, the other spins at 30 knots.

Figure 5 is an enlarged view of two targets with different movements, one linear and the other circular. This figure reinforces the tracking capacity of the system in different conditions of movement of the target. Furthermore, it attests the good choice of the dynamic turn-rate model for the targets. This choice is an essential factor for the success of the tracking system, as stated by [60].

**Figure 6:**
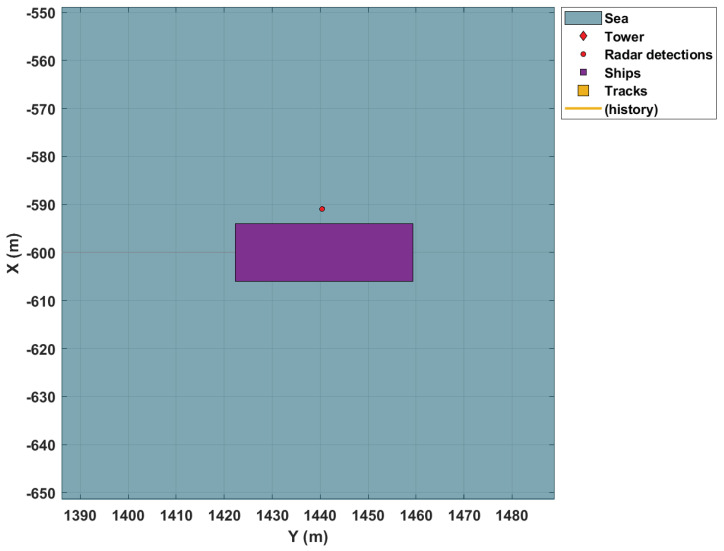
Target in occlusion.

Despite the successful choice for the prediction model, the figure illustrates an existing problem in real-life systems called target occlusion. Figure 6 shows this weakness when using only one radar. The larger vessel occludes the smaller one. Therefore, it is not detected by the tracking system. Given that the application aims to combat illegal activities usually carried out in the region by small vessels, it is essential to use sensors distributed throughout the surveillance area. This fact is further reinforced by the large dimensions of the surveillance area.

**Figure 7:**
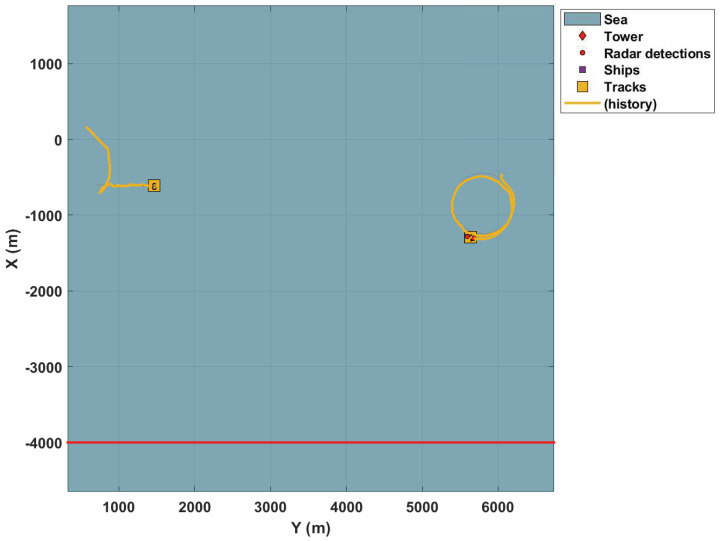
Second radar in the surveillance area.

Inserting a second radar prevents targets occlusion, as illustrated by Figure 7.

**Figure 8:**
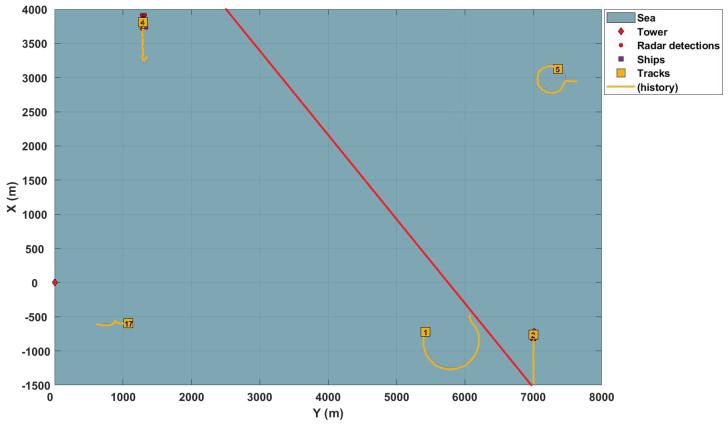
Complete tracking with merged PHDs.

**Figure 9:**
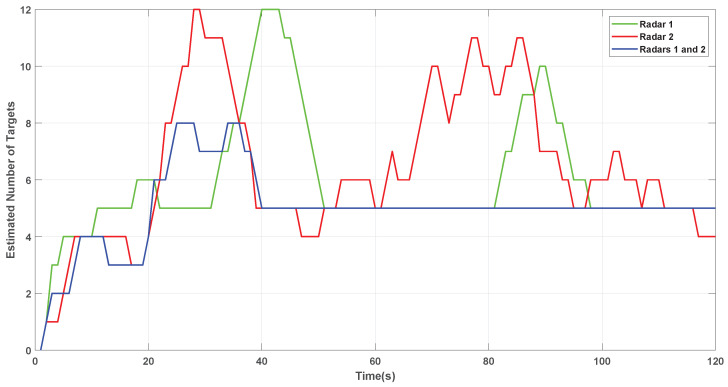
The number of estimated targets by individual trackers, and merged.

Figure 8 and Figure 9 present the results with combined trackers, i.e., two radars.

It is possible to observe that combining information from each tracker allows a faster convergence to the number of actual targets. In addition, it is more stable since the increase in data sources (sensors) in the system avoids targets occlusion compared to using only one radar.

**Figure 10:**
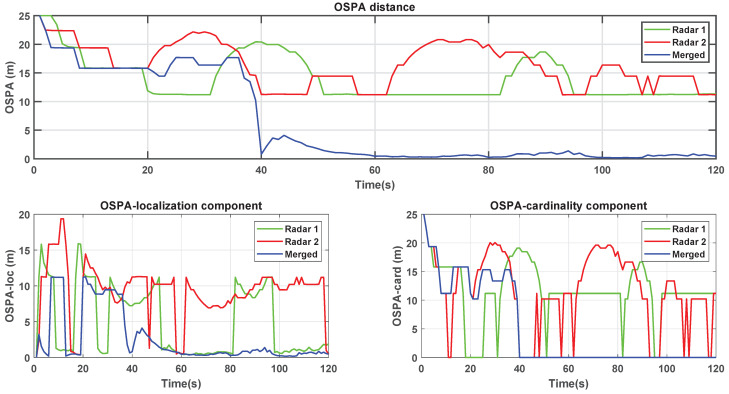
OSPA metrics.

Finally, Figure 10 presents the OSPA metric and its components, which corroborates the analyses based on the previous figures. The merged tracker is faster and more stable, as can be observed in the plots, in particular after a period of 40 s. The lower the metric value, the better the filter’s performance. Furthermore, the combined filter with data from both radars shows better results for localization and cardinality OSPA components. The location component is related to the position error, as can be seen in Figure 10, after 40 s, the value tends to zero. Similarly, at the same instant, the cardinality component becomes zero.

The simulations illustrate the problem of target occlusion when they present different geometries and dimensions. Inserting a new sensor and the consequent increase in the sensor network prevents targets occlusion and improves the overall performance of the tracking system. These results reinforce the statement by Vo and Ma [38]: The efficiency and simplicity in the implementation of the Gaussian mixture PHD recursion also suggest a possible application to tracking in sensor networks.

The algorithm has performance limitations already demonstrated in other researches. The proposed architecture presents good results when applying an additional step to the original algorithm, which is the main contribution of this work: A low-complexity data fusion scheme. Additionally, this proposed scheme presents promising results, especially when dealing with the target occlusion problem.

## 5. Conclusions

This paper introduces a Maritime Surveillance System for the Brazilian Coast. The system has a complex network of distributed sensors and processing stations. The demanding requirements for its operation are one of the motivations for the present work. In this context, this work presents an algorithm to track an unknown number of marine targets from multiple sensors in a large-scale surveillance area. A merged version of the Gaussian Mixture PHD filter is applied to a realistic multitarget tracking scenario. The proposed filter shows promising results through simulations, even for targets with different sizes, movements, and speeds. The proposed data fusion scheme improves the overall performance of the tracking system and overcomes targets’ occlusion. This fact further evidences, for the application, the need for a network of distributed sensors, given the system’s mission and the extension of the area of interest. Additionally, it is possible to conclude that increasing the covered area using sensors of a smaller capacity also contributes to the economic aspect of this modular undertaking.

For future work, one of the focuses would be investigating different RFS-based filters, the capabilities of the extended algorithms, and different sensor types such as stationary passive sonars or shipboard radars. Moreover, the computational models used for parallelism and multiprocessing should be investigated. In particular, those based on Gamma models, once SCUA data fusion applications already use this approach. These future works will attest, corroborate the empirical results obtained in the simulations, and contribute to using these algorithms in a new computational paradigm.

## Figures and Tables

**Table 1:** Radars and Central Station in simulation.

Entity	Coordinates [km]	Angular Sector Covered by FoV [rad]
Radar 1	(7.38, −0.236)	[0, −π/2]
Radar 2	(−4, 9)	[−π/2, π/2]
Central Station (Tower)	(0, 0)	Not Applicable

**Table 2:** Ships initial states and type of movement.

Ship	Initial Speed [knot]	Initial Orientation [Deg]	Initial Coordinates [km]	Type of Movement
1	20	130	(3.15, 7.4)	Circular with radius 200 m
2	30	120	(−0.5, 6)	Circular with radius 400 m
3	10	0	(3.2, 1.3)	Constant heading
4	12	0	(−1.5, 7)	Constant heading
5	6	90	(−0.6, 0.7)	Constant heading

**Table 3:** Filter parameters used for tracking targets.

Filter Parameter	Value
Sensor maximum range	12 km
Distance resolution noise	25 m
Azimuth resolution noise	0.5°
Probability of Survival (*p_S_*)	0.99
Probability of detection (*p_D_*)	0.9
Sensor field-of-view (FoV)	90°
Clutter density	2 × 10^−8^
Prune threshold (*T*)	10^−6^
Merge threshold (*U*)	25
Extraction threshold (*E*)	0.8
Maximum number of components	1000
